# Changing turn-over rates regulate abundance of tryptophan, GS biosynthesis, IAA transport and photosynthesis proteins in *Arabidopsis* growth defense transitions

**DOI:** 10.1186/s12915-023-01739-3

**Published:** 2023-11-09

**Authors:** Mohammad Abukhalaf, Carsten Proksch, Domenika Thieme, Jörg Ziegler, Wolfgang Hoehenwarter

**Affiliations:** 1 Present address: Institute for Experimental Medicine, Christian-Albrechts University Kiel, Niemannsweg 11, 24105 Kiel, Germany; 2 Department Biochemistry of Plant Interactions, Leibniz Institute of Plant Biochemistry, Weinberg 3, 06122 Halle (Saale), Germany; 3 Department Molecular Signal Processing, Leibniz Institute of Plant Biochemistry, Weinberg 3, 06122 Halle (Saale), Germany

**Keywords:** Protein synthesis rates, Protein degradation rates, Post-transcriptional regulation, PTI, Auxin, Pin proteins, Photosynthesis, MYC2, Glucosinolates, Phytohormone, Proteomics

## Abstract

**Background:**

Shifts in dynamic equilibria of the abundance of cellular molecules in plant-pathogen interactions need further exploration. We induced PTI in optimally growing *Arabidopsis thaliana* seedlings for 16 h, returning them to growth conditions for another 16 h.

**Methods:**

Turn-over and abundance of 99 flg22 responding proteins were measured chronologically using a stable heavy nitrogen isotope partial labeling strategy and targeted liquid chromatography coupled to mass spectrometry (PRM LC–MS). These experiments were complemented by measurements of mRNA and phytohormone levels.

**Results:**

Changes in synthesis and degradation rate constants (*K*_s_ and *K*_d_) regulated tryptophane and glucosinolate, IAA transport, and photosynthesis-associated protein (PAP) homeostasis in growth/PTI transitions independently of mRNA levels. *K*_s_ values increased after elicitation while protein and mRNA levels became uncorrelated. mRNA returned to pre-elicitation levels, yet protein abundance remained at PTI levels even 16 h after media exchange, indicating protein levels were robust and unresponsive to transition back to growth. The abundance of 23 PAPs including FERREDOXIN-NADP( +)-OXIDOREDUCTASE (FNR1) decreased 16 h after PAMP exposure, their depletion was nearly abolished in the myc234 mutant. FNR1 *K*_d_ increased as mRNA levels decreased early in PTI, its *K*_s_ decreased in prolonged PTI. FNR1 *K*_d_ was lower in *myc234*, mRNA levels decreased as in wild type.

**Conclusions:**

Protein *K*_d_ and *K*_s_ values change in response to flg22 exposure and constitute an additional layer of protein abundance regulation in growth defense transitions next to changes in mRNA levels. Our results suggest photosystem remodeling in PTI to direct electron flow away from the photosynthetic carbon reaction towards ROS production as an active defense mechanism controlled post-transcriptionally and by MYC2 and homologs. Target proteins accumulated later and PAP and auxin/IAA depletion was repressed in *myc234* indicating a positive effect of the transcription factors in the establishment of PTI.

**Supplementary Information:**

The online version contains supplementary material available at 10.1186/s12915-023-01739-3.

## Background

Plants are sessile organisms, and their life is characterized by the struggle to maintain a state of optimal growth and propagation, in a constantly changing biotic and abiotic environment. Interactions with the environment inevitably bring with them shifts to states of stress or alternative development or growth, characterized by shifting physiological dynamic equilibria.

Plant photoautotrophic growth is initially driven by photosynthesis and essentially composed of stem cell division and differentiation in the apical meristems followed by cell elongation [1]. It is orchestrated by a complex genetic program integrating diverse endogenous and environmental cues, including light, sugar, and temperature as well as phytohormone signaling entailing differential expression of thousands of genes. Auxin influences all aspects of plant development and growth, especially cell division and elongation [2]. Auxin/IAA is primarily synthesized from tryptophan by way of four pathways in *Arabidopsis thaliana*, utilizing the tryptophan metabolic products indole-3-acetaldoxime (IAOx), indole-3-acetamide (IAM), indole-3-pyruvic acid (IPyA), and tryptamine (TAM) as substrates, respectively [3]. It is synthesized in the shoot, root, and young leaf apical meristems and then transported from these regions of highest concentration throughout the plant by two transport systems, non-directional phloem stream and directional polar cell-to-cell transport (PAT) [3]. PAT is facilitated by membrane localized auxin efflux carriers of the PIN-FORMED family (PIN). PIN1 transports auxin in a basipetal direction from shoot to root whereas it is transported acropetally by PIN 2 [4]. The other canonical PINs PIN3, PIN4, and PIN7 transport the phytohormone away from tissue apices [5], making auxin patterning and establishment of local concentration gradients essential regulators of growth and developmental processes in all plant organs.

Plants have a decentralized immune system that induces pathogen associated molecular pattern (PAMP)/pattern recognition receptor (PRR) triggered immunity (PTI) upon perception of PAMPs by cell surface plasma membrane spanning receptors (PRRs) [6] such as FLAGELLIN SENSITIVE 2 (FLS2) binding the 22 amino acid N-terminal epitope of bacterial flagellin (flg22) [7]. Subsequent assembly of the mature, active receptor complex by recruitment of accessory receptor-like proteins (RLPs; BAK1 and others) [8, 9] and scaffolds (RK FERONIA family and others) [10] initiates Ca^2+^, ROS, and phosphorylation-mediated signaling, among others. All of these partially interconnected signaling relays ultimately lead to differential expression of thousands of genes [11] and extensive proteome remodeling [12]. Among others, PR proteins with anti-microbial activity [13] increase in abundance. Proteins in secondary metabolites/defense compounds synthesis pathways such as glucosinolates (GS) also increase in their abundance. Indolic glucosinolates (IGs) are synthesized from tryptophan [14] and upon cleavage by thioglucosidases called myrosinases give rise to a wide variety of toxic compounds such as isothiocyanates, thiocyanates, and nitriles [15, 16]. On the other hand, the proteins of the photosynthesis molecular machinery, including chloroplast thylakoid membrane integral photosystems II and I (PSII and PSI), are diminished in their abundance upon induction of PTI by flg22 [12, 17]. This is an active defense response, the meaning of which however is not fully understood. For excellent, current reviews of plant immunity, the reader is referred to [6, 18].

Salicylic acid (SA) and SA signaling are central and integrative components of both PTI and nucleotide-like receptor (NLR)/effector triggered immunity (ETI) [19]. Suppression of JA levels by SA in biotrophic pathogen resistance is well known [20]; however, a role of JA in PTI [21–24] and complementary activity of both hormones in fine tuning immunity to divergent pathogen lifestyles [25] have been documented, as well as numerous JA biosynthetic and signaling genes responding to flg22 [11]. The bioactive compound JA-Isoleucine (JA-Ile) produced by conjugation of isoleucine to JA by the jasmonate-isoleucine synthetase JAR1 is perceived by the SCF^COI1^–JAZ co-receptor complex, liberating expression of genes under the control of the JA signaling pathway. These include the basic helix-loop-helix (bHLH) transcription factor MYC2 and its homologs MYC3, MYC4, and MYC5, transcriptional activators that extensively orchestrate the genetic JA response program [26].

The JA signaling cascade and MYC2 activity are preeminent in the context of wounding and defense against biting insects, but studies in the past years have elucidated a function in the response to flg22 and immunity to biotrophic pathogens [21, 22, 27, 28]. MYC2 is a master regulator of gene expression and a central hub in the larger network that integrates phytohormone cross-talk and signaling [29]. It has been shown to bind to more than 6000 genes and more than 60% of all genes responding to exogenous JA application [30]. Furthermore, nearly half of 1717 known or predicted *Arabidopsis thaliana* TF genes were direct MYC2/3 targets and/or JA responsive. Also, the expression of many JA pathway genes, including JA synthesis itself, is under the control of MYC2 and MYC2 abundance and activity are extensively auto-regulated [31–34], evidence of positive and negative feedback control [35].

Cellular proteins undergo constant turn-over [36]. The protein pool is replenished by new synthesis and depleted by degradation of old proteins. The rate of synthesis is considered to follow zero-order kinetics, meaning it is independent of the amount of protein present. The degradation rate is modelled as first-order kinetics, meaning it depends on the amount of protein present in the pool over time. In physiological steady states of homeostasis, the two are in equilibrium and any changes in protein abundance are only due to growth. Shifts between steady states such as between growth and immunity, lead to changes in protein abundance due to proteome remodeling and perturb this equilibrium, resulting in potentially altered synthesis and degradation rates. Research in the last years have produced evidence that translational mechanisms play substantial roles in regulating plant immunity [37–39]. Changes in synthesis and degradation rates of proteins may be one such control mechanism.

Medium- to large-scale measurements of synthesis and degradation rates are possible combining a partial stable heavy isotope protein labeling strategy and mass spectrometry-based proteomics [40]. Under laboratory conditions, the organisms under study are switched to a stable heavy isotope containing diet as anabolic precursors at a given stage of development or growth. Incorporated into proteins, this leads to a shift in the peptide ion isotope envelope to greater mass to charge ratios (m/z) in liquid chromatography mass spectrometry (LC–MS) measurements of tryptic peptides over time. Increased signal intensity of higher-order isotope peaks is indicative of new protein synthesis due to incorporation of the heavy isotope into peptide primary structure whereas quenching of the naturally predominant monoisotopic peak is the result of protein degradation. Labeling proteins with the heavy nitrogen isotope ^15^N has proven to be the method of choice in plants [41] and has been demonstrated successfully in *Arabidopsis* [42], barley [43], and *Medicago truncatula* [44].

We developed a quantitative proteomics model of PTI comprising 99 proteins of the tryptophan, GS, JA biosynthesis pathways, members of the auxin biosynthesis pathways and phytohormone conjugation and metabolism, and auxin and JA signaling previously [12] as well as photosynthesis-associated proteins (PAPs) and proteins playing roles in primary metabolism (Additional file 1). Here we studied the chronology of the transitions between homeostasis and fully induced PTI steady states. Optimally growing plants were exposed to the PAMP flg22 for 16 h and then transferred back to optimal growth conditions free of the elicitor for the same amount of time. Two hundred thirty-one proteotypic peptides were targeted to measure synthesis and degradation rates (*K*_s_ and *K*_d_) of the 99 model proteins using a RT scheduled PRM scan strategy [45] as well as chronological changes in protein abundance in these state transitions. Each protein was monitored by at least 1 proteotypic peptide (Additional file 1). Transcript levels of 34 proteins from the same set were measured with an extra 4 proteins which were not amenable to MS measurements included CYTOCHROME P450 FAMILY 94 SUBFAMILY B POLYPEPTIDE 1 and 3 (CYP94B1 and CYP94B3), CYTOCHROME P450 FAMILY 94 SUBFAMILY C POLYPEPTIDE 1 (CYP94C1), and ISOCHORISMATE SYNTHASE 1 (ICS1) (Additional file 2). Moreover, we quantified the phytohormones abscisic acid (ABA), Indole-3-acetic acid (IAA), JA, JA-Ile, 12-hydroxy-JA (12-OH-JA), ( +)-12-oxo-phytodienoic acid (OPDA), and SA, which have important roles in growth and/or defense (Additional file 3). The same experiments were performed in the *myc234* knockout mutant to further explore the role of these transcription factors reported on previously [12].

## Results

### Changes in degradation and synthesis rate constants regulate shifts between growth and immunity

Protein *K*_s_ (synthesis) and *K*_d_ (degradation) rate constants were measured using a stable heavy nitrogen isotope partial labeling strategy [43]. After quality control filtering, *K*_d_ and *K*_s_ values under optimal growth/homeostasis conditions (C1) could be calculated directly from the chronological change in ^15^N incorporation (RIA, Additional file 4) for 68 of the 99 model proteins using Eqs. 2 and 3 and 5 and 6, respectively (Additional file 5). Thirty-three proteins degraded slowly including 11 of the 23 monitored PAPs (*K*_d_ < 0.055 day^−1^), 27 intermediately (*K*_d_ = 0.055–0.22 day^−1^), and 8 rapidly (*K*_d_ > 0.22 day^−1^) as described before [42].

**Fig. 1:**
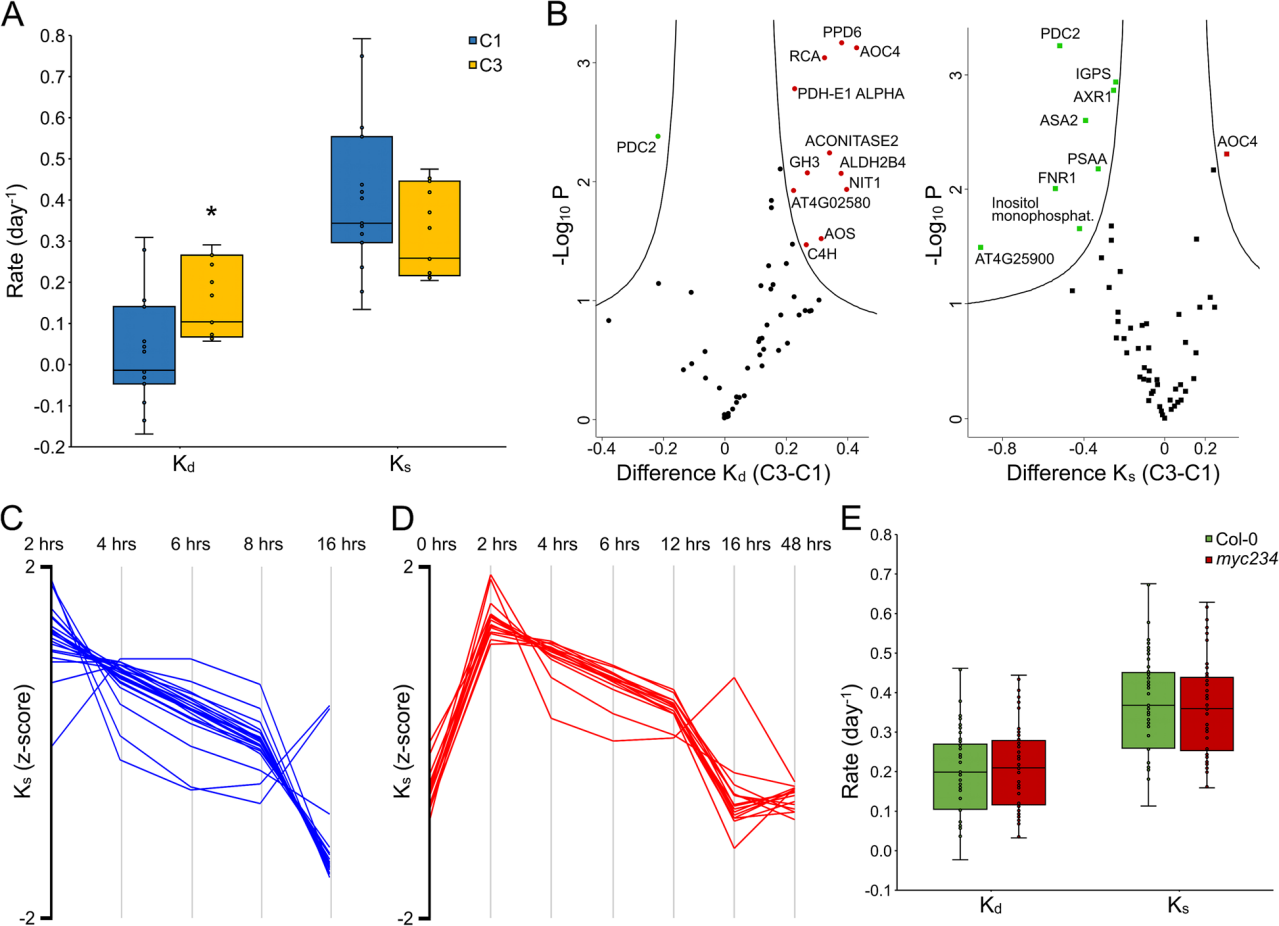
Post-transcriptional regulation of target protein abundance in flg22 induced PTI. **A** Synthesis (*K*_s_) and degradation (*K*_d_) rates of photosynthesis-associated proteins (PAPs) under conditions of homeostasis (C1) and fully induced PTI (C3). Star indicates statistically significant difference as determined by two-sided Student’s *t* test with *α* = 0.05 and *n* = 15. **B** Statistically significant differences in *K*_d_ and *K*_s_ values of all target proteins between conditions of fully induced PTI and homeostasis (Permutation-based FDR Student’s *t* test with *α* = 0.05, S0 = 0.1, and *n* = 3). **C** Chronological profile of protein *K*_s_ values in the establisher phase of PTI (C2) (*n* = 23). **D** Chronological profile of protein *K*_s_ values that were determined for all three conditions, homeostasis, establishment of PTI up to 16 h post exposure to flg22 and fully induced PTI (C1, C2, and C3) (*n* = 14). **E** *K*_s_ and *K*_d_ values of all targets in the Col-0 and *myc234* backgrounds in fully induced PTI (*n* = 53)

In a second experiment, flg22 was added to the culture medium (1 µM concentration in medium) at *t*_0_. The state transition from homeostasis to what we considered to be fully induced PTI at 16 h was termed condition 2 (C2) whereas steady-state PTI, from 16 to 96 h of PAMP exposure was termed condition 3 (C3). *K*_d_ and *K*_s_ values for C3 could be calculated for 69 proteins as for C1 (intersection C1 and C3 57 proteins) using *t*_0_, 8 h, and all later time points. Turn-over of 5, 31, and 33 proteins was slow, intermediate, and fast, reciprocal behavior as in steady-state growth suggesting immunity leads to an increase in metabolic activity. Of the 23 PAPs, 15 allowed calculations of *K*_d_ and *K*_s_ values in both C1 and C3. Degradation rate constants increased significantly for these proteins in steady-state PTI. Synthesis rate constants were lower but not significantly (Fig. 1A). This means that PAP abundance is post-transcriptionally controlled in PTI.

FERREDOXIN-NADP( +)-OXIDOREDUCTASE (FNR1), the terminal protein in the thylakoid electron transfer chain showed a significant decrease in its synthesis rate in prolonged PTI (Fig. 1B). FNR1 is a branching point in electron flow across the photosystems, transferring electrons derived from water oxidation onto NADP^+^ to produce NADPH which provides reducing potential for anorganic carbon assimilation in the stroma dark reactions for synthesis of high value organic poly-carbons. In its absence if photosynthetic linear electron transfer is disrupted and electrons are not transferred as readily onto NADP^+^, they are instead used to produce chloroplastic H_2_O_2_ (ROS) an integral part of plant defense signaling [46]. Other PAPs (Fig. 1B) with significant changes in their turn-over rate constants were the photosystem II oxygen evolving complex PsbP family protein PPD6 and the pyruvate dehydrogenase E1-alpha subunit (PDH-E1-Alpha) which were both degraded more rapidly. Pyruvate decarboxylase 2 (PDC2) which has been shown to render plants more tolerant to hypoxia [47] was turned-over more slowly in total (smaller *K*_d_ and *K*_s_ values). Inositol monophosphatase (AT5G64380) was synthesized more slowly.

C2 *K*_d_ values for 28 of the 99 proteins (Table 1) were calculated after quality control filtering with alternative Eq. 7 that incorporates the protein abundance fold changes over time, *K*_s_ values were calculated for 2, 4, 6, 8, and 16 h post flg22 exposure. Hierarchical cluster analysis showed that 23 of them had *K*_s_ values that changed over time and that were at a maximum 2 h after elicitation, decreasing over time and returning to their lowest levels 16 h after treatment with the PAMP (Fig. 1C). Moreover, *K*_s_ values of 14 of the 28 were calculated in all three conditions (C1, C2, and C3) and showed a strong increase after 0 h (C1), declining throughout the PTI establisher phase (C2) and returning to basal levels in fully induced PTI (C3) (Fig. 1D).

**Table 1:**
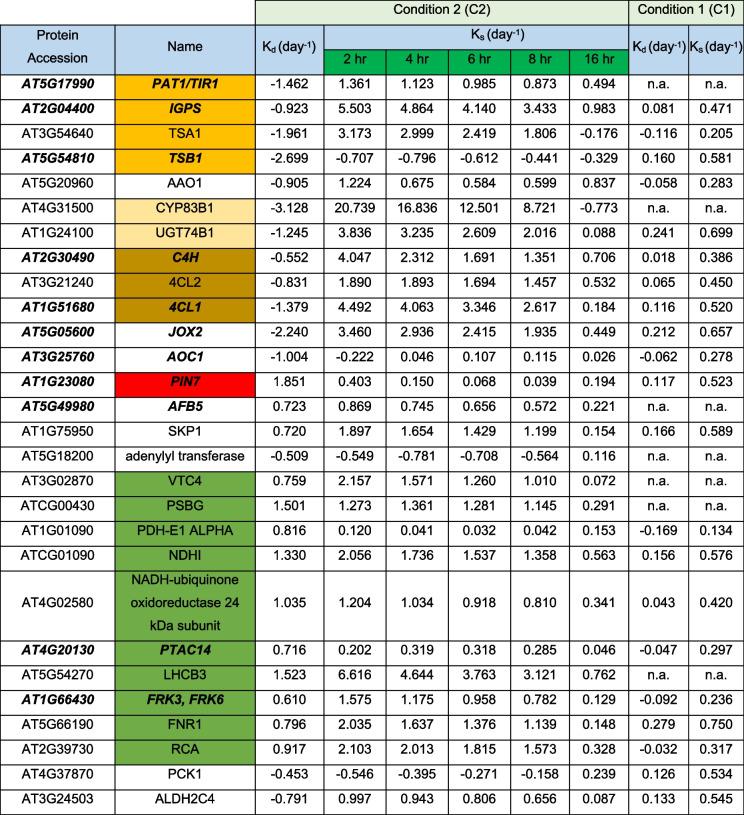
Protein synthesis (*K*_s_) and degradation (*K*_d_) rates under optimal growth conditions (C1) and the shift between growth and immunity (C2). Proteins in bold italics were also reported to be translationally regulated by [39]. Orange; tryptophan biosynthesis, Light orange, IG biosynthesis, Ochre, Phenylpropanoid biosynthesis, Green, PAP, Red, PIN-Protein

Of the 28 proteins *K*_d_ of 10 PAPs (Table 1 green color) increased, reflecting their degradation. This was also true for FNR1. *K*_d_ values of 9 proteins, belonging to tryptophan, GS, and phenylpropanoid biosynthesis pathways (Table 1 orange colors), decreased, even becoming negative, indicating the protein pool was stabilized, while at the same time *K*_s_ values increased markedly, indicative of synthesis of new protein, leading to the increase in the abundance of these proteins measured in the PRM experiments (below).

### Proteome is remodeled late and remains stable into PTI recovery phase back to growth

**Fig. 2:**
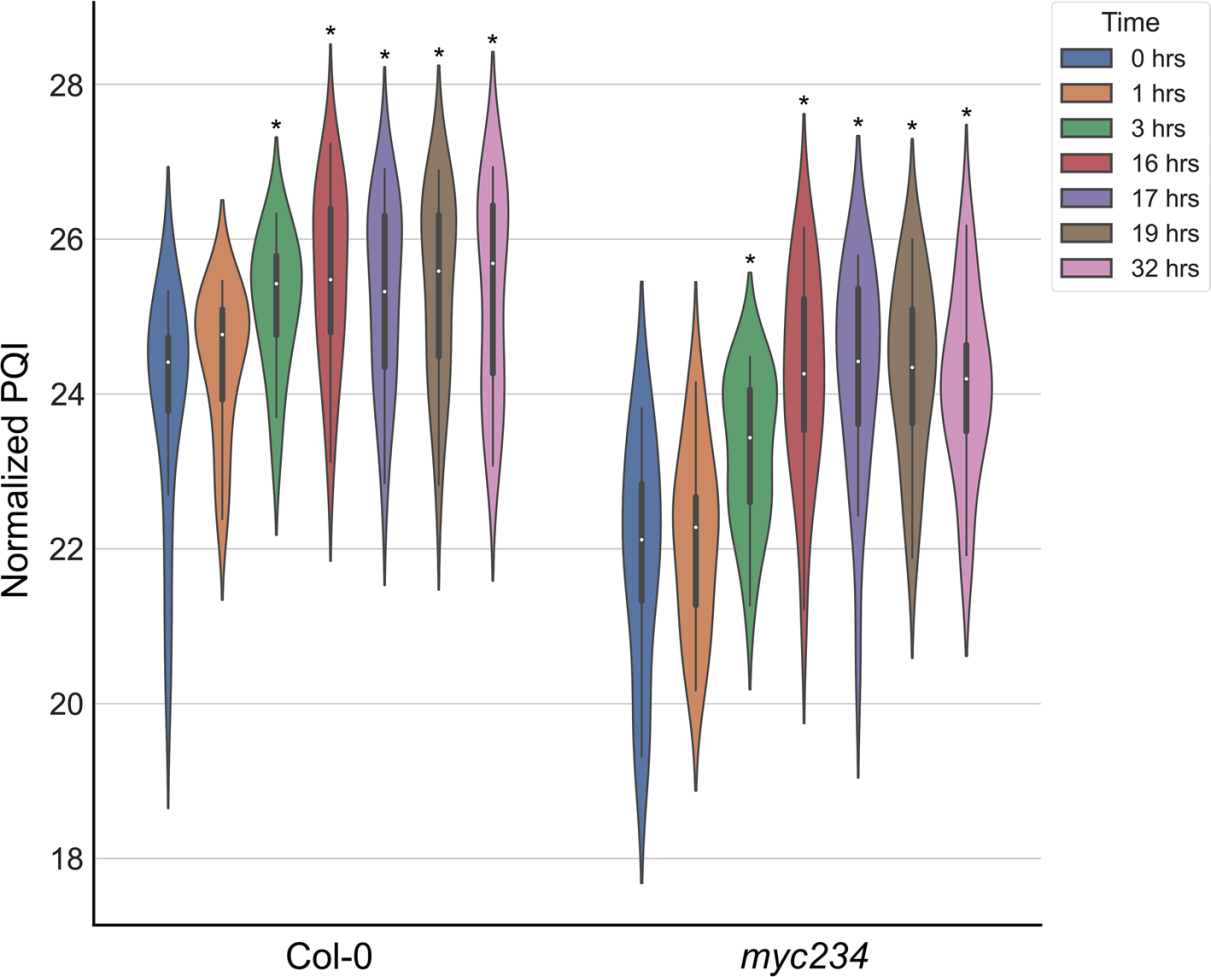
Violin plot of LOG_2_ median area under the curve (AUC) values of targeted proteotypic peptides for each protein (protein quantification indices, PQI). PQI values for tryptophan, GS, and other secondary metabolites biosynthesis proteins are plotted (*n* = 18) for the different experimental sampling points. Box represents 1st and 3rd quartiles, tip of lines the maximum and minimum (excluding outliers), and the white dot is the median. Kernel probability density distributions are superimposed. Stars indicate statistically significant differences in respect to the 0 h time point as determined by two-sided Student’s *t* test with *α* = 0.05

Target protein abundance was measured by PRM throughout the experiment (Additional file 6). Tryptophan biosynthesis pathway protein levels, PHOSPHORIBOSYLANTHRANILATE TRANSFERASE 1 (PAT1), INDOLE-3-GLYCEROL PHOSPHATE SYNTHASE (IGPS), TRYPTOPHAN SYNTHASE ALPHA CHAIN (TSA1), and TRYPTOPHAN SYNTHASE BETA-SUBUNIT 1 (TSB1), increased at 1 and 3 h and peaked at more than double their steady-state growth levels 16 h after PAMP exposure. The same trend was observed for proteins of the GS, phenylpropanoid, and 4-OH-ICN biosynthesis pathways (Fig. 2). Tryptophan channels into both IG and auxin synthesis and these pathways are known to be induced upon exposure to flg22 [14]. Interestingly, protein abundance remained elevated after transfer back to flg22 free medium until the last sampling time point at 32 h. This was contrary to our expectations of a return to optimal growth levels as the plants recovered from fully induced immunity.

**Fig. 3:**
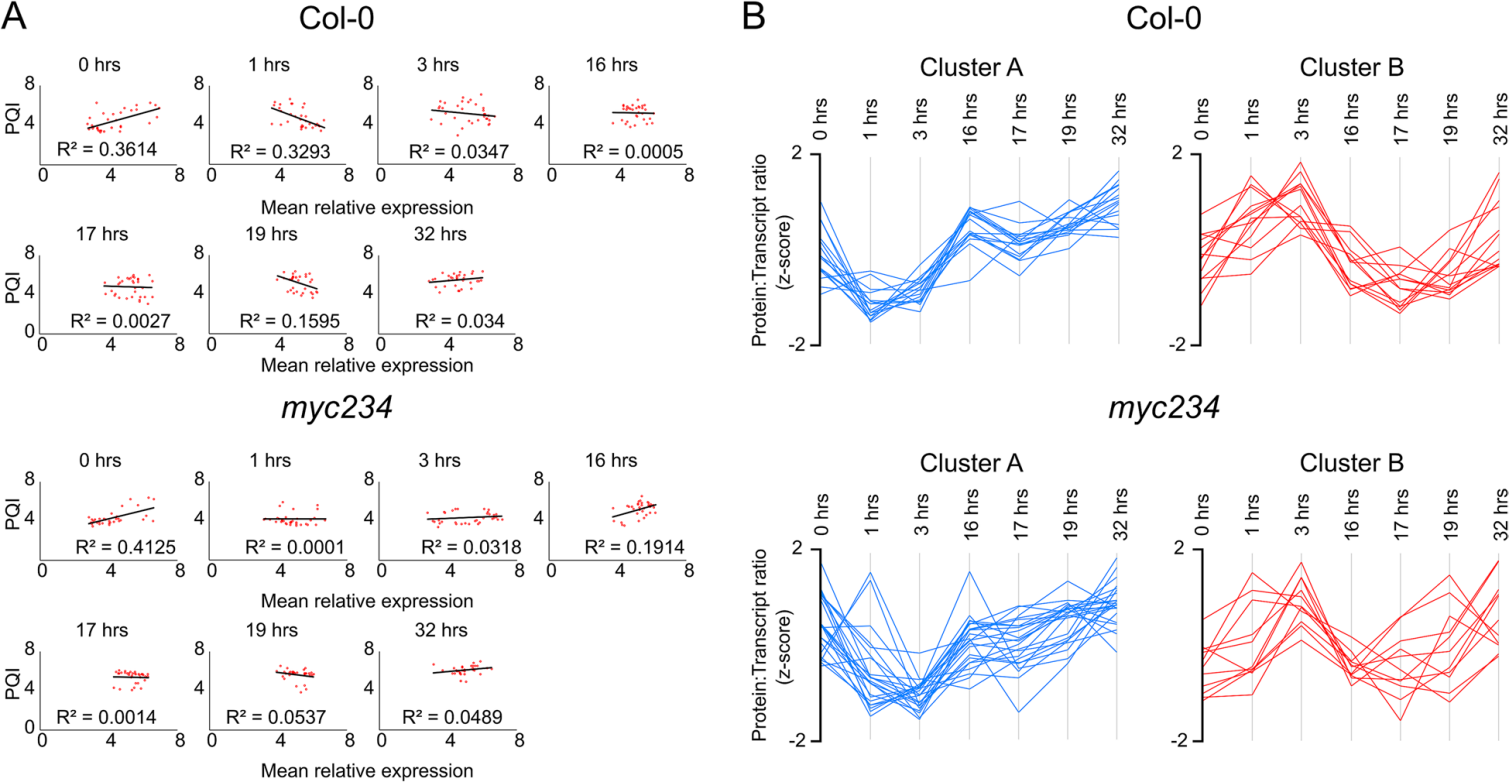
**A** Linear correlation between mRNA (independent variable) and protein abundance (dependent variable) at all sampling time points in wild type and *myc234*. Mean relative expression and PQI values were log_10_ and *Z*-score transformed and increased by 5 (*n* = 34). **B** Protein to transcript abundance ratios (log_10_ and *Z*-score transformed PQI and mean relative expression increased by 5) throughout experimental sampling time points. Col-0. Cluster A (*n* = 16); Cluster B (*n* = 13). *myc234* background. Cluster A (*n* = 23); Cluster B (*n* = 11)

Transcript levels of 34 target proteins (Additional file 7) showed a positive linear correlation (R^2^) of 0.36 to protein abundance in growth conditions which became negative after 1 h of exposure to flg22 and deteriorated almost completely after 3 and 16 h (Fig. 3A). It remained negative over all PTI time points, indicating transcription of cognate genes occurs early in the state transition and that there is a considerable lag time before translation. Transcripts returned to initial growth levels after 16 h of transfer to flg22 free medium as opposed to proteins.

Protein to transcript ratios of the 34 proteins (Additional file 7) at the experimental sampling time points produced two major clusters (Fig. 3B, Additional file 8). Sixteen ratios decreased to a minimum 1 and 3 h after flg22 exposure then increased well above values in unchallenged plants at the 16 h sampling point where PTI is fully induced (cluster A) again meaning transcript abundance increased early followed by translation. Ratios remained elevated throughout the recovery phase, indeed increasing further at the 32 h sampling point where we expected optimal growth to have been reestablished, showing that despite degradation of transcripts, cognate proteins remained stable even 16 h into the transition to optimal growth.

Thirteen protein to transcript ratios (cluster B) increased 1 and 3 h after challenge with the PAMP followed by a decrease to a minimum at 16 h post flg22 treatment returning to growth levels after removal of the PAMP (Fig. 3B, Additional file 8). This indicated early decrease in transcript followed by protein abundance several hours later for this set, suggesting post-transcriptional control mechanisms regulating protein abundance in growth PTI transitions in addition to mRNA levels. They also suggest that proteins accumulating under conditions of pathogen resistance especially those involved in defense compound synthesis remain abundant in the cell and that degradation and clearance of these proteins, even upon return to optimal growth, may not be expedient or necessary.

Also, *K*_s_ and mRNA levels both increased early after PTI induction at the outset of proteome remodeling. Synthesis rates are determined by many parameters including mRNA levels; however, the correlations (R^2^) between *K*_s_ and mRNA levels for 13 proteins for which we measured mRNA levels were 0.03, 0.01, and 0.04 at 1, 3, and 16 h post PAMP exposure, respectively (Additional file 8). This shows that in our experiment the two were largely independent and that other factors primarily determined the synthesis rate itself.

### Downregulation of photosynthesis in PTI

**Fig. 4:**
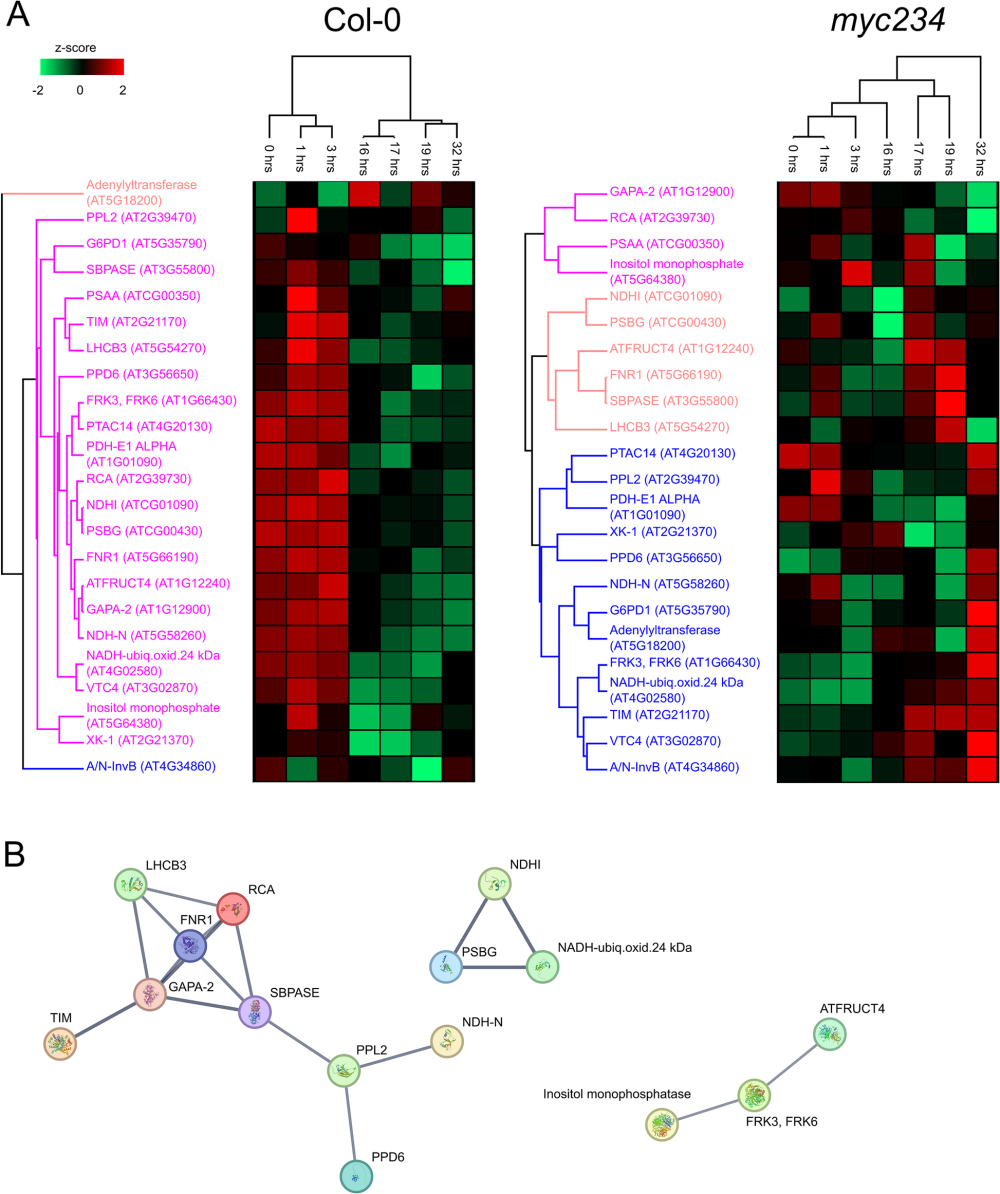
**A** Hierarchical clustering of *Z*-score transformed LOG_2_ fold changes in protein abundance (FCP) in respect to the 0 h time point of 23 PAPs in Col-0 and *myc234*. **B** Full STRING network of the 23 PAPs (line thickness indicates confidence, interaction sources include experiments, databases and co-expression, minimum interaction score of 0.7 (High confidence))

Twenty of the 23 PAPs including photosystem I and II (PSI and PSII) components that are primary orchestrators of the light reaction, Calvin cycle proteins, and proteins playing roles in photorespiration were significantly less abundant 16 h after exposure to flg22 in the wild type (Fig. 4A, Additional file 6). Protein levels remained low throughout the transition back to optimal growth up until the 32 h sampling point. Inhibition of photosynthesis has been reported previously as an active part of the plant immune response to pathogens [46]. FNR1 transcripts were diminished to nearly 0.5 fold 3 h after PAMP exposure and remained at approximately this level throughout the remaining sampling time points (Additional file 2). Protein levels decreased to − 0.6 log_2_ fold change 16 h after exposure and remained at this level or slightly lower throughout the rest of the experiment (Additional file 6).

PAPs interacted physically and/or functionally (Fig. 4B) including members of PSII and the oxygen evolving complex such as PPD6, which had a higher *K*_d_ value in PTI. NDH complex and associated proteins (NDH-N, NDHI PPL2, PSBG) also diminished in abundance, suggesting reduced activity of cyclic electron transport, another potential acceptor of electrons from ferredoxin. Other interactors were dark reaction proteins such as RCA and SBPase, whose activity has been shown to directly influence photosynthetic capacity and plant growth [48]. The results suggest depletion of FNR1 and other PAPs leads to active remodeling of both the thylakoidal and stromal photosynthetic apparatus to drive electron transfer into ROS production and switch its function from growth to defense.

### Polar auxin transport may play an important role in shifts between growth and immunity

**Fig. 5:**
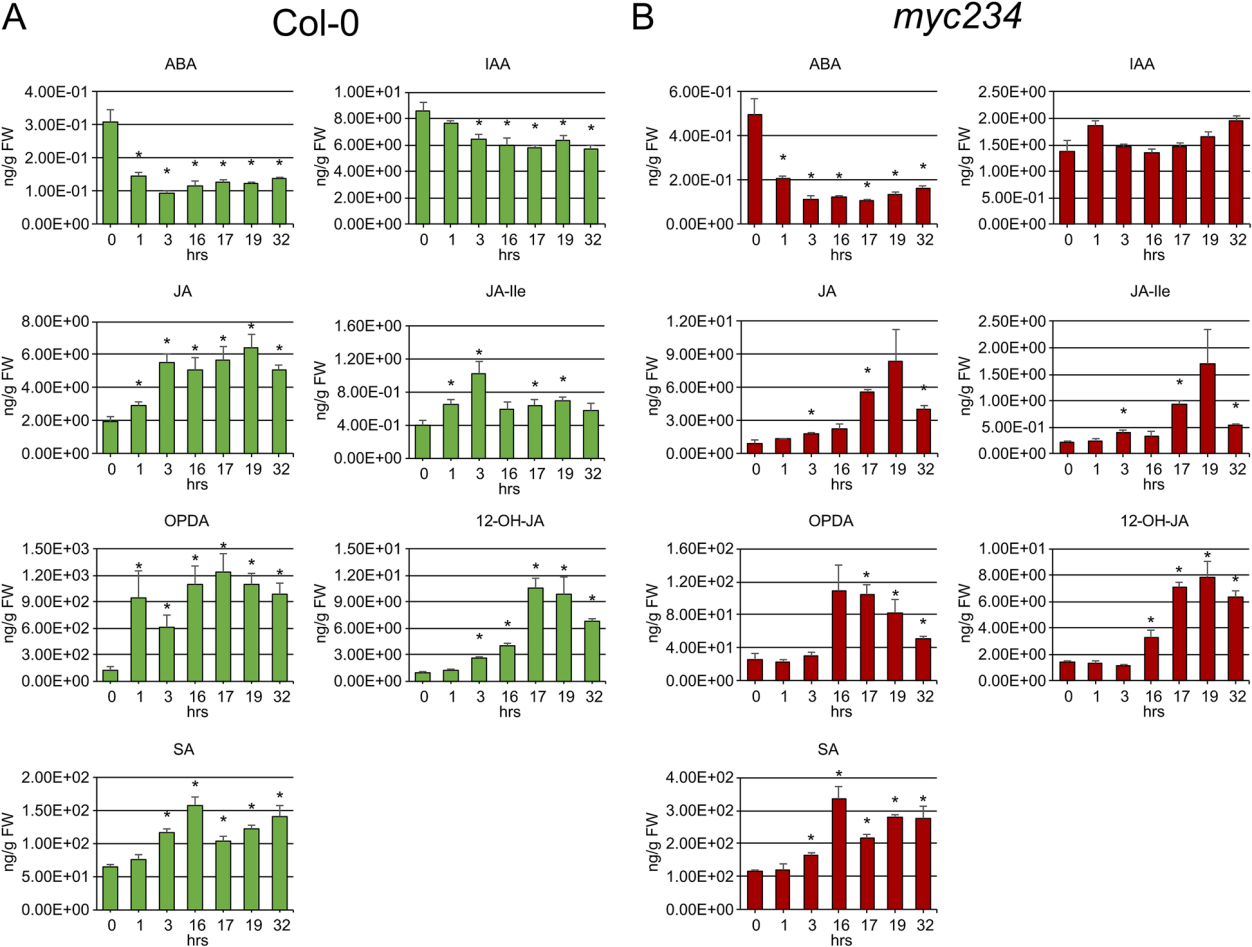
MRM-based quantification of phytohormone levels throughout experimental sampling time points in Col-0 (**A**) and *myc234* (**B**) backgrounds. Error bars denote standard error (SE). Stars indicate statistically significant differences in respect to the 0 h time point as determined by two-sided Student’s *t* test with *α* = 0.05 and *n* = 4 or 3 (Col-0 or *myc234*)

The abundance of several proteins playing roles in auxin transport and auxin and JA signaling reached a minimum at 16 h post induction of PTI or 1 and 3 h post transfer to flg22 free medium yet returned to growth levels 16 h after transfer to fresh medium (32 h time point). The auxin efflux carriers PIN3 and PIN7, which are primarily responsible for polar cell-to-cell auxin transport, showed log_2_ fold changes of − 1.06 and − 1.45 16 h after flg22 treatment and min log_2_ fold changes of − 1.28 and − 1.88 1 h after medium switch, respectively. Their abundance then returned to initial levels throughout the state transition back to growth. As also reported previously [12], global auxin levels significantly decreased by around 20% at *α* = 0.05, 3, and 16 h post PTI induction and at the 17, 19, and 32 h sampling points into the recovery phase (Fig. 5, Additional file 3). Together, this suggests auxin transport and localization may play an important role in shaping PTI, in addition to reduction of the total amount of auxin/IAA itself. The PIN7 *K*_d_ value increased markedly, underscoring the importance of post-transcriptional regulatory processes.

### Delayed PTI onset, reduced auxin levels, and attenuated depletion of photosynthesis proteins in myc234 mutant

The same set of experiments as in the wild type was performed in the *myc234* triple knockout mutant [49]. Protein *K*_d_ or *K*_s_ values did not change significantly after long-term induction of PTI (C3) (Additional file 9, Fig. 1E), transitionary phase (C2) *K*_d_ values of PAPs which could be calculated in the *myc234* background including FNR1, were well below wild type (Table 2). The abundance of tryptophan, GS, 4-OH-ICN, and phenylpropanoid biosynthesis pathway proteins was reduced, and their accumulation delayed in the mutant becoming pronounced at the 3 and 16 h time points whereas it was already higher 1 h after exposure to the PAMP in Col-0 (Fig. 2, Additional file 6). Many of the genes in these pathways are targets of MYC2 and MYC3 [30], and it has been reported that their expression in the triple mutant is reduced [50].

**Table 2:** Protein degradation rates of PAPs that were measured in both Col-0 and *myc234* backgrounds in the establisher phase (C2) up to 16 h post flg22 perception

Accession number	Protein name	*K*_d_ (day ^−^ ^1^) Col-0	*K*_d_ (day ^−^ ^1^) *myc234*
AT3G02870	VTC4	0.759	0.094
AT1G01090	PDH-E1 ALPHA	0.816	0.576
ATCG01090	NDHI	1.330	0.617
AT4G02580	NADH-ubiquinone oxidoreductase 24 kDa subunit	1.035	− 0.163
AT5G66190	FNR1	0.796	0.524

The correlation between transcript and protein levels was 0.41 in optimal growth and similar to the wild type throughout the experiment (Fig. 3A). It remained positive throughout the growth PTI transition, albeit with a smaller slope, indicating a later increase of transcripts and accumulation of proteins in the mutant (Additional file 7). Protein to transcript ratios clustered as in wild type, 87.5% of cluster A protein to transcript ratios in the wild type were also cluster A members in the mutant, 69% of cluster B members related in this way, including the PIN3 and PIN7 auxin transporters (Fig. 3B, Additional file 8). Notable exceptions that showed discordant cluster membership were AAO1, JAR1, COI1, and TPR1, proteins involved in auxin synthesis, auxin and JA conjugation, and JA perception and signaling. JA synthesis, conjugation, and signaling are auto-regulated by positive and negative feedback loops dependent on MYC2 [31–34].

Interestingly, downregulation of PAP abundance was nearly abolished in the *myc234* mutant background (Fig. 4A), implying a role of these transcription factors in impingement of photosynthetic activity in the response to PAMP treatment. It is known that transcript levels of photosynthesis and growth-related genes as well as leaf growth are repressed upon coronatine treatment, an analog of JA [51]. Together our experiments in the triple mutant suggest a positive role of MYC2 in the establishment of PTI, especially in regulation of the function of the photosystem.

### Phytohormone levels in the growth PTI state transitions

In the wild type and mutant, JA and JA-Ile levels remained at nearly basal levels, increasing slightly but significantly at 1 and 3 h post flg22 exposure (Fig. 5, Additional file 3). SA levels increased significantly after 3 h and remained elevated during the transition to growth in the wild type. Substantially more SA was measured in the mutant, significantly accumulating to higher levels throughout the experiment. The opposite was true for ABA, whose abundance decreased significantly in both genetic backgrounds already after 1 h of exposure to the PAMP and remained at approximately the same level at all sampling points. Auxin/IAA was less abundant in the *myc234* mutant background and levels did not change significantly throughout the experiment as in the wild type suggesting the moderate depletion upon PTI elicitation is dependent on MYC2 and homologs (Fig. 5).

## Discussion

We chose 99 targets that showed a strong flg22 response previously [12] to investigate regulation of protein abundance by changes in synthesis and degradation rates. Transcriptomics studies employing flg22 [11, 52] or Met-JA treatment [30, 53] used varying exposure times from 1 to 16 h and [52] showed that flg22 marker gene expression generally returned to basal levels between 12 and 24 h, which we also found. The 16-h recovery phase in flg22 free medium was an arbitrary choice because to our knowledge no studies of this kind had been performed previously.

Both basal and induced immunity, phytohormone biosynthesis, and other metabolic and physiological processes are attuned to a 24-h time cycle by the circadian clock, a central, essentially free running, genetic oscillator [54–57]. Our experiment uncoupled clock and immunity as there was no change in the protein abundance during the day in the absence of PAMP (see Additional file 10 [12, 58–61]). Concerns that physical manipulation of the seedlings during the transfer between media may have elicited a JA response were similarly unfounded.

The observed positive correlation (R^2^) of around 0.4 between protein and mRNA abundance under growth conditions in both the wild type and *myc234* mutant attributes 40% of protein abundance to cognate mRNA levels. This value finds general consensus in the literature, but correlation can vary considerably depending on organism, tissue, and biological context [62, 63]. A study in maize uncovered divergent protein to transcript abundance ratios in the developmental zones of the same leaf blade that were highly dependent on protein function in the respective zones [64]. Another work in *Arabidopsis* described the protein to mRNA abundance correlation becoming negative as plants shifted from cold acclimation to de-acclimation [65] which the authors attributed to storage of nascent transcripts followed by subsequent translation on demand. Changes in protein abundance are a question of protein turn-over, underlying both new synthesis and degradation from the current pool. Our study provides *K*_s_ and *K*_d_ values for our targets in both growth and PTI steady states as well as the transition between states and shows that they change in response to flg22. Eleven of the 28 proteins with changes in protein turn-over in the transition shown in Table 1 were also reported to be translationally regulated in response to flg22 by Tabassum and co-workers measuring relative changes in translated mRNA using Ribo-seq [39] whereas only 3 also responded to elf18 [37]. Thus, our results and others indicate post-transcriptional regulatory processes determine changes in protein abundance in the cell in plant immunity in addition to changes in mRNA levels. A study in human cell lines [66] also noted that changes in protein synthesis rates are primary determinants of protein abundance in transitions between physiological steady states. The correlation between *K*_s_ values and mRNA levels, where available in our experiments, was negligible.

Transcripts and proteins comprising the tryptophan and aliphatic and indolic glucosinolate (IG) biosynthesis pathways increased in their abundance after exposure to flg22. The latter are important defense compounds required for callose deposition in PTI, and the elevated expression of IG biosynthesis genes has been described previously in response to flg22 [14]. Tryptophan is the metabolic precursor channeling into IG biosynthesis and we link induction of its synthesis to flg22 perception here and in our previous study [12]. We did not measure IG levels themselves because Clay et al. [14] showed that cleavage by the myrosinases PEN2 and PEN3 to activate them hampers direct measurement.

Induction of both tryptophan and GS biosynthesis pathways has been reported to be an integral part of JA-mediated defenses in response to wounding, necrotrophic pathogens, herbivores, exogenous application of JA, and constitutive JA signaling in a *jazD* mutant devoid of ten of the family members of the JAZ repressor proteins [50, 67–71]. Introgression of a *myc234* loss of function mutation into the *jazD* background abolished expression of biosynthetic pathway genes and accumulation of tryptophan and IGs [70] and the *myc234* mutant is known to be compromised in both tryptophan and GS biosynthesis [50]. This agrees with our measurements of basal levels of these biosynthesis pathway proteins in the triple mutant below wild type levels. Interestingly however, protein levels increased in the mutant as in wild type upon flg22 treatment in our experiment although at 3 h as opposed to 1 h after exposure, suggesting that in PTI, tryptophan and GS biosynthesis does not depend on MYC2 and homologs and indeed, MYB51 downstream of ethylene signaling mediates the elevated expression of IG biosynthesis genes in PTI [14]. Our experiments suggest context-dependent signaling activity through MYC2 and that multiple, genetically independent pathways control GS biosynthesis.

Tryptophan is also a metabolic precursor of auxin/IAA and overexpression of TRYPTOPHAN SYNTHASE BETA subunit (TSB) genes from broccoli in *Arabidopsis* led to accumulation of both auxin and GS [72]. Indole-3-acetaldoxime (IAOx) is the metabolic precursor of both [73] as well as camalexin [74] in *Arabidopsis* and this compound and metabolic flux have been discussed as possible modulators of the growth defense trade-off. However, this opinion has not solidified and is not supported by our measurements. Despite the induced expression of tryptophan, IG, and camalexin biosynthesis pathway transcripts and proteins and a general increase in tryptophan itself [12], auxin levels were affected only slightly upon flg22 treatment, decreasing by about 20% throughout the experiment. Thus, a major decrease in total auxin levels does not seem to accompany PTI to affect the reduction in seedling growth which we measured upon PAMP exposure. Conspicuously, a reduction in total free auxin/IAA content was not observed in the *myc234* mutant background. *myc2* single loss of function mutants *jin1-9* and *jin1-10* also exhibited elevated free auxin levels [75] supporting our results that MYC2 negatively regulates these. The authors attributed this suppressive effect to the inhibition of tryptophan biosynthesis and so to reduced availability of IAA metabolic precursors.

Most tryptophan, GS, and camalexin biosynthesis pathway transcripts returned to basal levels at the end of the recovery phase of the experiment, yet cognate protein levels remained elevated even 16 h after shift of the plants to flg22 free medium. It is known that proteome remodeling is energetically costly and that the proteome is more stable and conserved than the transcriptome [76] and adjustment of the abundance of these proteins may not immediately be necessary for a physiological shift back to homeostasis. Also, the perpetuated elevation may be attributed to a type of priming phenotype where the plant is prepared to deal with future attackers upon initial activation of PTI [77].

The abundance of the polar auxin transport proteins PIN3 and PIN7 decreased early and continuously over the period of flg22 exposure yet increased again rapidly, returning to basal levels when the PAMP was removed. A decrease in the abundance of these proteins and perturbed auxin distribution has been reported in response to *Alternaria brassica* infection [68]. The decrease in their abundance in PTI establishment and the return to initial levels in the transition back to growth suggests, that local auxin gradients may be more important in regulating growth defense shifts than global auxin levels per se. PIN3 and PIN7 expression is not downstream of JA signaling in response to flg22 as in the immunity to necrotrophic pathogens or xylem development [68] because protein abundance levels in the *myc234* mutant were similar to wild type.

Downregulation of the expression of photosynthesis-associated genes (PAG) and photosynthetic activity under numerous biotic and abiotic stress as well as exogenous application of phytohormones has been amply documented ([51, 78] and citations therein). A reduction in the abundance of cognate proteins has been shown here and by us and others previously [12, 17]. Two recent reports disclosed these phenomena and concurrent production of chloroplast reactive oxygen species (cROS) as part of the active plant defense response to a wide range of bacterial effectors [79, 80]. Two types of cROS are generated, singlet oxygen (^1^O_2_) by transfer of excitation energy from triplet-state Chl in PSII [81] and hydrogen peroxide (H_2_O_2_) by electron transfer and oxygen reduction (Mehler reaction) from ferredoxin in PSI [82]. cROS act as defense signaling molecules that affect a wide range of pathways including the MEP pathway to stimulate defense hormone signaling and gene expression [83]. Depletion of photosynthesis-associated proteins (PAP) particularly PSI and PSII components and inhibition of photosynthesis were a prerequisite for cROS production, suppression of PTI, and robust induction of ETI in response to pathogen effectors [79, 80]. Blocking of electron transfer from PSII by 3-(3,4- dichlorophenyl)-1,1-dimethylurea (DCMU) led to inhibition of H_2_O_2_ production at PSI and compromised immunity against *P. syringae* DC3000 *hrpA*, a disarmed pathogen unable to deliver effectors into the cell and elicit ETI [79].

We have measured the reduced abundance of PAPs in response to the PAMP flg22 repeatedly, although this was excluded by SU and co-workers [80]. Presumably, this was because the moderate fold changes we observed escaped detection by the SDS-PAGE technique that they used to visually show downregulation of PSI and PSII proteins. We also link the diminished abundance of PAPs in flg22 elicited PTI to MYC2 activity because it was severely diminished in the *myc234* mutant background. Attaran and co-workers [51] reported depletion of PAPs in response to exogenous application of Met-JA but only transient inhibition of photosynthetic activity resulting from a lack of CO_2_. This agrees with upheld electron transfer to PSI. Current models [46] postulate electron transfer along the thylakoid membrane in PTI leading to H_2_O_2_ production at PSI whereas extensive depletion of PAPs leads to its interruption and more vigorous ^1^O_2_ production in ETI.

FNR1 is the final protein in the electron transfer chain that has the reducing potential to transfer an electron from ferredoxin onto NADP^+^ producing NADPH. If this reaction does not proceed because of a lack of FNR1 and the electron is not funneled into cyclic electron transport and ATP production because of absence of NDH complex components, which we also observed, it instead goes into oxygen photoreduction and H_2_O_2_ production [79]. The *fnr1 x fnr2* heterozygote displayed reduced photosynthetic activity and increased ion leakage indicative of increased ROS production [84]. We measured a significant reduction in the protein’s synthesis rate in PTI elicited by the PAMP (C3) and an elevation in its degradation rate in the earlier establisher phase of PTI (C2), which was dependent on MYC2 and homologs, concurrent with a decrease in FNR1 transcript and protein abundance upon flg22 exposure. Our results suggest reshaping of the photosynthetic protein apparatus as an active defense response mediated by MYC2 and homologs in PTI as opposed to the MPK3/MPK6-mediated mechanism disclosed for ETI [80]. In this model, certain key components such as NDH complex components and FNR1 act as molecular switches between growth and immunity.

Our experiments in the *myc234* mutant uncovered a positive role of these bHLH transcription factors in the early establisher phase of PTI and potentially in defense against biotrophic pathogens. MYC2 and homologs are canonical JA responsive transcription factors and some of the effects we observed were also observed downstream of JA signaling. However, it may be noted that throughout our entire experiment JA and JA-Ile were hardly elevated above basal levels (low ng/g FW range) despite significant increases in fold change. These phytohormones increase around a 100-fold of what we measured in *Arabidopsis* response to wounding [85] and insect herbivory [86]. This raises the question if MYC2, MYC3, and MYC4 may be targets of another signaling pathway in *Arabidopsis* flg22 induced PTI. Liu and co-workers disclosed degradation of JAZ repressor proteins and MYC2 activation by the SA receptors NPR3 and NPR4 upon *Pseudomonas syringae* pv *maculicola* ES4326 with *avrRpt2* but not with the same strain devoid of the effector [87]. This was evidence of non-canonical MYC2 activation in the ETI context.

## Conclusions

Translational regulation by way of changes in protein synthesis and degradation rate constants adds another level affecting protein abundance in PTI in addition to changes in mRNA levels. The latter are chronologically discordant with fold changes in the abundance of cognate proteins. Our results shed light on an emerging picture of complex regulatory relationships that integrate hormone and other signaling pathways and transcriptional and post-transcriptional control that is temporally and context dependent to fine-tune protein expression and expedite growth defense shifts. Depletion of key PAP components seems to be an active defense response to remodel the entire photosynthetic apparatus to direct electrons gained from the light-dependent oxidation of water into ROS as opposed to NADPH production. Thus, these key proteins may be instrumental in modulating the growth defense shifts and indeed the trade-off between the two in plants.

## Methods

### Measurement of protein degradation and synthesis rates

*Arabidopsis thaliana* Col-0 and *myc234* triple knockout mutant seedlings were grown in liquid cultures. Two hundred fifty *Arabidopsis thaliana* seeds were grown in 50 ml 25% MS medium (0.748 mM CaCl_2_, 0.313 mM KH_2_PO_4_, 0.375 mM MgSO_4_, 4.7 mM KNO_3_, 5.15 mM NH_4_NO_3_, 2.35 mM MES buffer, 0.25 × Basal Salt Micronutrients solution, 0.25 × Vitamin solution, and 2.5 g/L sucrose, pH 5.7) on an orbital shaker 45 rpm at 22 °C under long day conditions (16 h light, 8 h dark) for 10 days. Cultures were harvested in triplicate, representing the unlabeled population, time point was designated as *t*_−8_. Remaining cultures were transferred to 25% MS medium containing ^15^N as the sole nitrogen source (K^15^NO_3_ and ^15^NH4^15^NO_3_) and grown further under the same conditions for 8 h to ensure incorporation of the ^15^N label into primary structure for LC–MS measurement. This was designated as *t*_0_ and the start of the experiment. Culture fresh weights were recorded at *t*_−8_, *t*_0_, and 1, 2, 4, 16, 28, 40, 64, and 88 h into the experiment.

To measure protein synthesis and degradation rates under PTI conditions, flg22 was added to a concentration of 1 µM in medium at *t*_0._ Cultures were sampled at this time point in triplicate and further sampled 2, 4, 6, 8, 16, 24, 32, 48, 72, and 96 h after flg22 injection. Harvested tissue was weighed and frozen at − 80 °C.

Proteins were extracted and samples prepared for MS analysis as described [12]. Ion peak isotope envelopes of proteotypic target peptides of the 99 cognate target proteins were measured with the PRM strategy also described in [12]. Raw data were converted to.MzML format using msconvert [88]. Protover, a python-based code program, was used to extract the relative isotopic abundance (RIA) of ^15^N in peptide ion isotope envelopes over all sampling time points [89] (Eq. 1, Additional file 4).

1 $$RIA = \frac{15N}{15N+14N}$$

where 15N represent the heavy and 14N the naturally abundantly occurring isotope population.

The list of proteotypic target peptides with their average retention times gained from the PRM experiments was used as an input for the program. A retention time window of 5 min was set as filter and the “RIA increasing” filter was disabled. The program output which consisted of target peptides and their RIA in all measurements of all replicates at all sampling time points were further analyzed using Excel. Only peptides with RIA values at 5 or more time points in at least one replicate were retained. Then median RIA values of cognate proteotypic peptides were used to infer RIA values for respective proteins at all sampling time points in each replicate.

Under optimal growth conditions the apparent degradation rate (*K*_loss_) was calculated for each protein from the slope of the logarithmic function -ln(1-RIA) plotted over time as in Eq. 2.

2 $${K}_{loss}*t = -\mathrm{ln}(1-RIA)$$

where *t* is time in days.

Then proteins with relative standard errors (RSTDE) of *K*_loss_ values of the three replicates greater than 40%, median R^2^ values below 0.5 and proteins with negative *K*_loss_ values were discarded. The actual degradation rate constant (*K*_d_) for each protein was then calculated using Eq. 3.

3 $${K}_{d}= {K}_{loss}- {K}_{dil}$$

where *K*_dil_ is the doubling constant.

*K*_dil_ was calculated by fitting an exponential model to the weight increase of seedlings over experimental sampling time points shown in Additional file 10; Supplementary figure 1 as a proxy for the increase of total protein amount over time as in Eq. 4.

4 $${A}_{t}= {A}_{0}* {e}^{{K}_{dil}*t}$$

where *A*_*t*_ is the total amount of proteins at time (*t*) and *A*_0_ is the proteins amount at *t* = 0.

To calculate the synthesis rate constant (K_s_/A), Eq. 5 [42] was used.

5 $$\frac{{K}_{s}}{A}=\frac{FCP-{e}^{-{K}_{d}*t}}{1-{e}^{-{K}_{d}*t}}*{K}_{d}$$

where FCP is Fold Change of Protein Abundance.

By reorganizing Eq. 4, FCP can be calculated as follows:

$$FCP=\frac{{A}_{t}}{{A}_{0}} = {e}^{{K}_{dil}*t}$$

By substitution in Eq. 5 (K_s_/A) can be calculated as in Eq. 6.

6 $$\frac{{K}_{s}}{A}=\frac{{e}^{{K}_{dil}*t}-{e}^{-{K}_{d}*t}}{1-{e}^{-{K}_{d}*t}}*{K}_{d}$$

A mean time of 2 days was used to calculate *K*_s_/*A* for all filtered proteins (later as *K*_s_).

Protein *K*_s_ and *K*_d_ values were calculated separately for the transitionary phase between optimal growth and fully induced PTI at 16 h post flg22 exposure and fully induced PTI itself, 16 h and beyond. For the early establisher phase, only time points 0, 2, 4, 6, 8, and 16 h after flg22 treatment were used. To calculate the *K*_d_, the slope of the logarithmic function in Eq. 7 was used:

7 $${K}_{d}*t = -\mathrm{ln}(FCP*\left(1-RIA\right))$$

However, here FCP was estimated at the respective time points by fitting a polynomial second-order model to the PRM data protein abundance measurements using median FCP at time points 0, 1, 3, and 16 h. *K*_d_ and *K*_s_ values were only calculated for proteins with FCPs higher than 1.5 or lower than 0.75. Values, model equations and correlation (R^2^) measures are given in Additional file 11. The mean correlation was 0.95 indicating high quality of fit for all proteins. The same estimated PRM data FCPs were used for all biological replicates. Median peptide RIA values were used to infer cognate protein RIA values for *K*_d_ calculations and proteins with measured RIA values at 4 time points or more in at least one biological replicate were considered. Proteins with relative standard errors (RSTDE) of *K*_d_ values of the three replicates (TSA1 and AAO1 had only two replicates) equal to or less than 40% and median R^2^ values of 0.5 or more were considered. For calculation of *K*_d_ values in fully induced PTI, Eqs. were used as above using time points 0, 8, 16, 24, 32, 48, 72, and 96 h after flg22 treatment in the *K*_loss_ and *K*_dil_ calculations. *K*_s_ values in both phases were calculated using Eqs. 5 and 6 as above.

### Experimental design and statistical rationale

To model transitory shifts between optimal growth and PTI and back, *Arabidopsis* seedlings were grown in liquid culture for 10 days and then supplemented with flg22 (1 µM in medium). Plants were sampled in triplicate at the time of induction, 1, 3, and 16 h post induction, followed by transfer of plants back to flg22 free medium and sampling 1, 3, and 16 h post transfer (later referred to as 17, 19, and 32 h). Statistical power analysis for a one-sided *t*-test was done to calculate the minimum number of samples *n* to achieve 80% power at a significance level *α* of 0.05. The effect size expressed as Cohen’s *d* was estimated as the median *d* of all target peptides in a previously performed PRM experiment quantifying changes in target protein abundance which generally increased after 16 h of flg22 exposure published in [12]. LC–MS PRM experiments were performed where triplicates were injected twice for a total of 6 measurements (*n* = 6; three biological replicates with two technical each) of each sampling point.

### PRM, qPCR, and phytohormone measurements

PRM LC–MS measurements were performed in a blinded fashion. Interference by co-eluting peptides or other ionized compounds with identical m/z tolerating a 5 ppm difference was assayed by checking target peptide extracted ion current chromatograms and matching peak apices RTs to RTs for MS2 identifications provided by Proteome Discoverer. Plant cultivation, growth, treatment, sample preparation, targeted PRM LC–MS measurements, peptide and protein identification, PRM-based area under the curve (AUC) peptide, and protein quantification can be found in Additional file 10 and were performed as described previously in Document S1 of [12] with no modifications. In brief, target peptides were quantified by extracting MS2 ion chromatograms from the PRM measurements and calculating area under the curve (AUC) values of fragment ion peak envelopes over chromatographic RT. mRNA isolation, cDNA synthesis, qPCR (Additional file 12) and phytohormone extraction, sample preparation and MRM LC–MS phytohormone measurements were also performed as described therein with no modifications.

### Supplementary Information

**Additional file 1.** List of proteotypic target peptides (PTPs).

**Additional file 2. **qPCR data of target proteins in Col-0 and *myc234*.

**Additional file 3. **SRM LC-MS of phytohormones in Col-0 and *myc234*.

**Additional file 4. **Relative isotopic abundance (RIA) in partial labeling experimens in Col-0 (untreated and exposed to flg22) and *myc234* (exposed to flg22).

**Additional file 5. **Protein K_d_ and K_s_ values in Col-0 (untreated and flg22 exposed).

**Additional file 6. **Fold changes in protein abundance (FCP) in Col-0 and *myc234*.

**Additional file 7. **Protein to transcript ratios in Col-0 and *myc234*.

**Additional file 8. **Protein to transcript ratio cluster members in Col-0 and *myc234*.

**Additional file 9. **Protein K_d_ and K_s_ values in *myc234* (flg22 exposed).

**Additional file 10. **Supplemental methods, experiments and figures.

**Additional file 11. **Model fitting of FCPs in Col-0 including model equation and quality of fit.

**Additional file 12. **List of qPCR primers.

**Additional file 13. **LC-MS measurements of flg22 in growth medium.

**Additional file 14. **FCPs in Col-0 untreated.

## Data Availability

All raw and metadata have been deposited to ProteomeXchange Consortium via the Pride partner repository with the dataset identifier PXD041215 and can be found at: http://www.proteomexchange.org [90].

## Abbreviations

12-OH-JA: 12-Hydroxy Jasmonic Acid
4CL1: 4-Coumarate:CoA Ligase 1
4CL2: 4-Coumarate:CoA Ligase 2
AAO1: Aldehyde Oxidase 1
ABA: Abscisic acid
ACO2: ACC Oxidase 2
ACX1: Acyl-CoA Oxidase 1
AOC1: Allene Oxide Cyclase 1
AOC2: Allene Oxide Cyclase 2
AOC4: Allene Oxide Cyclase 4
AOS: Allene Oxide Synthase
ASA2: Anthranilate Synthase 2
AUC: Area under the curve
AUX1: Auxin Resistant 1
AXR4: Auxin Resistant 4
BAK1: BRI1-Associated Receptor Kinase 1
bHLH: Basic Helix-Loop-Helix
BIK1: Botrytis-Induced Kinase 1
C4H: Cinnamate 4-Hydroxylase
CDPKs: Calcium-Dependent Protein Kinases
COI1: Coronatine Insensitive 1
CUL1: Cullin 1
CYP81F2: Cytochrome P450, Family 81, Subfamily F, Polypeptide 2
CYP83A1: Cytochrome P450, Family 83, Subfamily A, Polypeptide 1
CYP83B1: Cytochrome P450, Family 83, Subfamily B, Polypeptide 1
CYP94B1: Cytochrome P450, Family 94, Subfamily B, Polypeptide 1
CYP94B3: Cytochrome P450, Family 94, Subfamily B, Polypeptide 3
CYP94C1: Cytochrome P450, Family 94, Subfamily C, Polypeptide 1
EDS5: Enhanced Disease Susceptibility 5
EIN3: Ethylene Insensitive 3
ETI: Effector Triggered Immunity
FCP: Fold Change Protein
FDR: False discovery rate
flg22: Flagellin 22
FLS2: Flagellin Sensitive 2
FNR1: Ferredoxin-NADP ( +)-Oxidoreductase 1
GS: Glucosinolates
IAA: Indole-3-Acetic Acid
IAR3: IAA-Alanine Resistant 3
ICS1: Isochorismate Synthase 1
IGPS: Indole-3-Glycerol Phosphate Synthase
JA: Jasmonic acid
JA-Ile: Jasmonic acid isoleucine
JAR1: Jasmonate Resistant 1
JAZ: Jasmonate ZIM Domain
JOX2: Jasmonic Acid Oxidase 2
*K*_d_: Degradation rate
*K*_dil_: Growth rate (dilution factor)
*K*_loss_: Apparent degradation rate
*K*_s_: Synthesis rate
LAX: Like AUXIN1
LHCB3: Light-Harvesting Chlorophyll B-Binding Protein 3
LOX2: Lipoxygenase 2
MAPKs or MPKs: Mitogen Activated Protein Kinases
MeJA: Methyl Jasmonate
NINJA: Novel Interactor Of JAZ
NIT4: Nitrilase 4
NTL9: Nac Transcription Factor-Like 9
OPDA: 12-Oxo-phytodienoic acid
OPR3: Oxophytodienoate-Reductase 3
PAD4: Phytoalexin Deficient 4
PAL1: Phenylalanine Ammonia-Lyase 1
PAMPs: Pathogen Associated Molecular Patterns
PAT: Polar Auxin Transport
PAT1: Phosphoribosyl Anthranilate Transferase 1
PDH-E1 ALPHA: Pyruvate Dehydrogenase E1 Alpha
PEN2: Penetration 2
PIN3: PIN-Formed 3
PIN7: PIN-Formed 7
PP2A: Protein Phosphatase 2A Subunit A3
PQI: Protein Quantification Index
PRM: Parallel Reaction Monitoring
PRRs: Pattern Recognition Receptors
PTI: Pattern Triggered Immunity
RBOHD: NADPH OXIDASE RESPIRATORY BURST OXIDASE HOMOLOG
RIA: Relative Isotopic Abundance
ROS: Reactive Oxygen Species
RSTDE: Relative Standard Error
SA: Salicylic acid
SCF^COI1^: Coronatine Insensitive1 Skp1/Cullin/F-Box Complex
SKP1: S Phase Kinase-Associated Protein 1
SOT16: Sulfotransferase 16
SUR1: Superroot 1
TCP22: TCP Domain Protein 22
TFs: Transcription Factors
TIM: Triosephosphate Isomerase
TPL: Topless
TPR: Topless-Related
TSA1: Tryptophan Synthase Alpha Chain
TSB1: Tryptophan Synthase Beta-Subunit 1
UBC21: Ubiquitin-Conjugating Enzyme 21
UGT74B1: UDP-Glucosyl Transferase 74B1
UGT74F2: UDP-Glucosyltransferase 74F2
