# Supplementary material for: Changing turn-over rates regulate abundance of tryptophan, GS biosynthesis, IAA transport and photosynthesis proteins in Arabidopsis growth defense transitions
Source: BMC Biol. 2023 Nov 9;21:249. doi: 10.1186/s12915-023-01739-3 (PMC10634109; doi:10.1186/s12915-023-01739-3)
Supplement: Supplementary file 10 — Additional file 10. Supplemental methods, experiments and figures. [file 12915_2023_1739_MOESM10_ESM.pdf]

Supplemental Methods, Experiments and Figures

Protein extraction and digestion

Extraction and digestion were done as previously described (12) . 1 mL of an extraction buffer (4% SDS, 50 mM Tris buffer, 10 mM EDTA, 10 mM DTT, 1.5% Protease inhibitor cocktail for plants(Sigma-Aldrich), pH 8.0) was added to 500 mg of ground seedlings powder and mixed at 95°C for 10 min, vortexed for 30 secs and then mixed at 22°C for 20 min. The extracts were centrifuged at 16,000 g for 10 min at 10°C and supernatants were transferred to new tubes and centrifuged again at 20,000 for 30 min at 10°C. Protein concentrations were determined by a 2D-Quant kit (Cytiva)

Proteins were digested according to the FASP protocol. After adding 100 µg proteins to Amicon® Ultra 30K filters 0.5 mL (Merck), samples were washed three times with UA (8 M Urea, 50 mM Tris buffer, pH 8.0) by centrifugation at 16,100 for 10 min at 22°C. Samples were then reduced by 100 mM DTT in UA for 1 hr at 22°C then after centrifugation alkylated by 50 mM IAA in UA for 1 hr at 22°C in the dark. After that, samples were washed again three times with UA, three times with ABC (50 mM Ammonium bicarbonate, pH 8.0) and digested by adding Lys-C (200:1) for 4 hrs at 37°C then by adding Trypsin (50:1) and left overnight at 37°C. Digested peptides were collected by centrifugation as before then by adding 40 µL ABC and centrifugation again. Pooled peptides were vacuum dried and desalted with in-house packed C18 tips (six layers C18 matrix in 100 µL tips).

LC-MS analysis of proteins

Proteotypic peptides (PTPs) for each of the 99 target proteins were selected iteratively wherein the 3 PTPs with the highest number of peptide spectral matches and Mascot ion scores of each target protein identified in data dependent (DDA) LC-MS measurements of total protein extracts of untreated and flg22 treated (16 hrs) seedlings were taken and their

m/z used to populate the peptide target list. Proteins for which 3 DDA measured PTPs were unavailable were *in silico* digested using d::ppop (58) and the peptides with the top predicted ESI response were targeted using a targeted data acquisition (TDA) scan strategy. Identified PTPs and their m/z were added to the peptide target list. Thirdly, PTPs which were not picked up in either DDA or TDA measurements were targeted using a PRM scan strategy. Lastly, retention times and retention time windows for each PTP m/z on the peptide target list were added and fit to produce the final PRM target list containing the m/z of the 231 PTPs each of which was unique to a single protein. Extracted ion current (EIC) chromatograms of each PTP m/z were manually inspected to ensure there was no interference by ion signals of peptides with similar m/z (m/z window +/- 5 ppm).

To control possible PTM of target peptides following flg22 perception which would potentially introduce errors in quantification, DDA measurements of 16 hrs flg22 treated samples were searched tolerating serine and threonine phosphorylation as variable PTMs. 187 of the 231 unmodified target peptides were identified in the DDA experiments without any cognate phosphopeptide identification for any one of them, indicating essentially no measurable PTM of target peptides after induction of PTM.

Dried peptides were dissolved in 5% acetonitrile, 0.1% trifluoroacetic acid, and injected into an EASY-nLC 1000 liquid chromatography system. Peptides were separated using liquid chromatography C18 reverse phase chemistry employing a 180 min gradient increasing from 5% to 40% acetonitrile in 0.1% FA, and a flow rate of 250 nL/min. Eluted peptides were electrosprayed online into a Q ExactiveTM Plus mass spectrometer (Thermo). A full MS survey scan was carried out with chromatographic peak width set to 15 s, resolution 35,000, automatic gain control (AGC) 1E+06 and a max injection time (IT) of 100 ms. The full scan was followed by retention time scheduled PRM scanning without multiplexing with HCD fragmentation. MS/MS scans were acquired with resolution 17,500, AGC 2E+05, IT 100 ms, loop count 10, isolation width 1.6 m/z, isolation offset 0.5 and a normalized collision energy 27.

Peptides and proteins were identified using the Mascot software v 2.5.0 (Matrix Science) (59) linked to Proteome DiscovererTM v 2.1 (Thermo). The enzyme was set to trypsin. A precursor ion mass error of 5 ppm and a fragment ion mass error of 0.02 Da were tolerated in searches of the TAIR10 database amended with common contaminants (35934 sequences, 14486974 residues). Carbamidomethylation of cysteine was set as a fixed modification and oxidation of methionine (M) was tolerated as a variable modification. A PSM, peptide and protein level false discovery rate (FDR) was calculated for all identified spectra and peptides and proteins based on the target-decoy database model. The significance threshold α was set at 0.01 to accept PSM, peptide and protein identifications. Quantitative analysis of PRM data was done with the Skyline software v 19.1.0 (60) . A spectral library was created using all measurements. The sum of six picked product ion signal peak areas was extracted as (Protein Quantification Index) PQI. PQI values were normalized by multiplication with a factor for each sample based on TIC maximum intensity.

Measurement of flg22

Media samples at time points 0, 1, 16, 17 and 19 hrs. were filtered with Amicon® Ultra 30K filters. Filters were washed overnight in 5% Tween-20 and then three times with ddH_2O for 30 minutes each. 300 µL of the media sample were added to the filter and then centrifuged at 16,100 g for 10 min at RT. The filtrates were kept and dried in a vacuum concentrator and the resulting dried samples were desalted on STAGE-Tips C18. The final dried peptide samples were dissolved in 136 µL 5% ACN, 0.1% TFA to reach a final concentration of 2.2 µM flg22 for LC-MS analysis.

Dried peptides were dissolved in 5% acetonitrile, 0.1% trifluoroacetic acid, and injected into an EASY-nLC 1000 liquid chromatography system. Peptides were separated using liquid chromatography C18 reverse phase chemistry employing a 60 min gradient increasing from 5% to 40% acetonitrile in 0.1% FA, and a flow rate of 250 nL/min. Eluted peptides were electrosprayed on-line into a Q Exactive^{TM} Plus mass spectrometer. The spray voltage was

1.9 kV, the capillary temperature 275°C and the Z-Lens voltage 240 V. A full MS survey scan (DDA top10) was carried out with chromatographic peak width set to 15 s, resolution 70,000, automatic gain control (AGC) 3E+06 and a max injection time (IT) of 100 ms. MS/MS scans were acquired with resolution 17,500, AGC 5E+04, IT 50 ms, loop count 10, isolation width 1.6 m/z, isolation offset 0.0 and a normalized collision energy 28.

Flg22 peptides were identified using the Mascot software v 2.5.0 (Matrix Science) linked to Proteome DiscovererTM v2.1. The enzyme was set to none. A precursor ion mass error of 5 ppm and a fragment ion mass error of 0.02 Da were tolerated in searches of the flg22 sequence as a database. Oxidation of methionine (M) was tolerated as a variable modification. A PSM, peptide and protein level false discovery rate (FDR) was calculated for all identified spectra and peptides and proteins based on the target-decoy database model. The significance threshold α was set to 0.01 to accept PSM, peptide and protein identifications.

Experimental design validation

We wanted to control that no flg22 was transferred with the seedlings meaning the recovery phase back to homeostasis was clear of elicitor. To this end we quantified flg22 in growth media at sampling time points 0, 1, 16, 17, and 19 hrs (Supplemental Fig 2) (Additional file 13). As expected flg22 was not detected after transfer of the seedlings, however, we were surprised to find that one hour after introduction to the medium full length flg22 accounted for only 9% of all identified flg22 derived peptides (breakdown products) and after 16 hours the full-length peptide was depleted completely and only three products with 10, 11 and 12 amino acids remained. Nonetheless, these flg22 breakdown products contain the core epitope recognized by FLS2 and thus presumably were still active, though less efficiently (61).

To ensure that the physical transfer did not elicit changes in protein abundance, we performed an experiment in which plants were mock treated with water and samples were

taken at 0, 16, 17 and 19 hrs in quadruplicate. We quantified our target proteins and analyzed the results using hierarchal clustering (Supplemental Fig 3A). Both row and column dendrograms showed random clustering. Proteins produced two clusters, the major cluster containing 92 of the 99 target proteins (Supplemental Fig 3B). Additionally, $\log_2$ protein fold changes of sampling time points in respect to time point 0 were tested for significance using a one sample T-test (FDR multiples testing corrected $\alpha=0.01$) with the null hypothesis H_0 being no changes in protein abundance across all time points (Additional file 14). Only one protein (PAL1) showed a significant increase in abundance at the 16 hrs sampling point all together indicating that neither physical transfer of plants or other experimental factors including the time of day of sampling or amount of light had affected protein abundance.

Supplemental figure 1

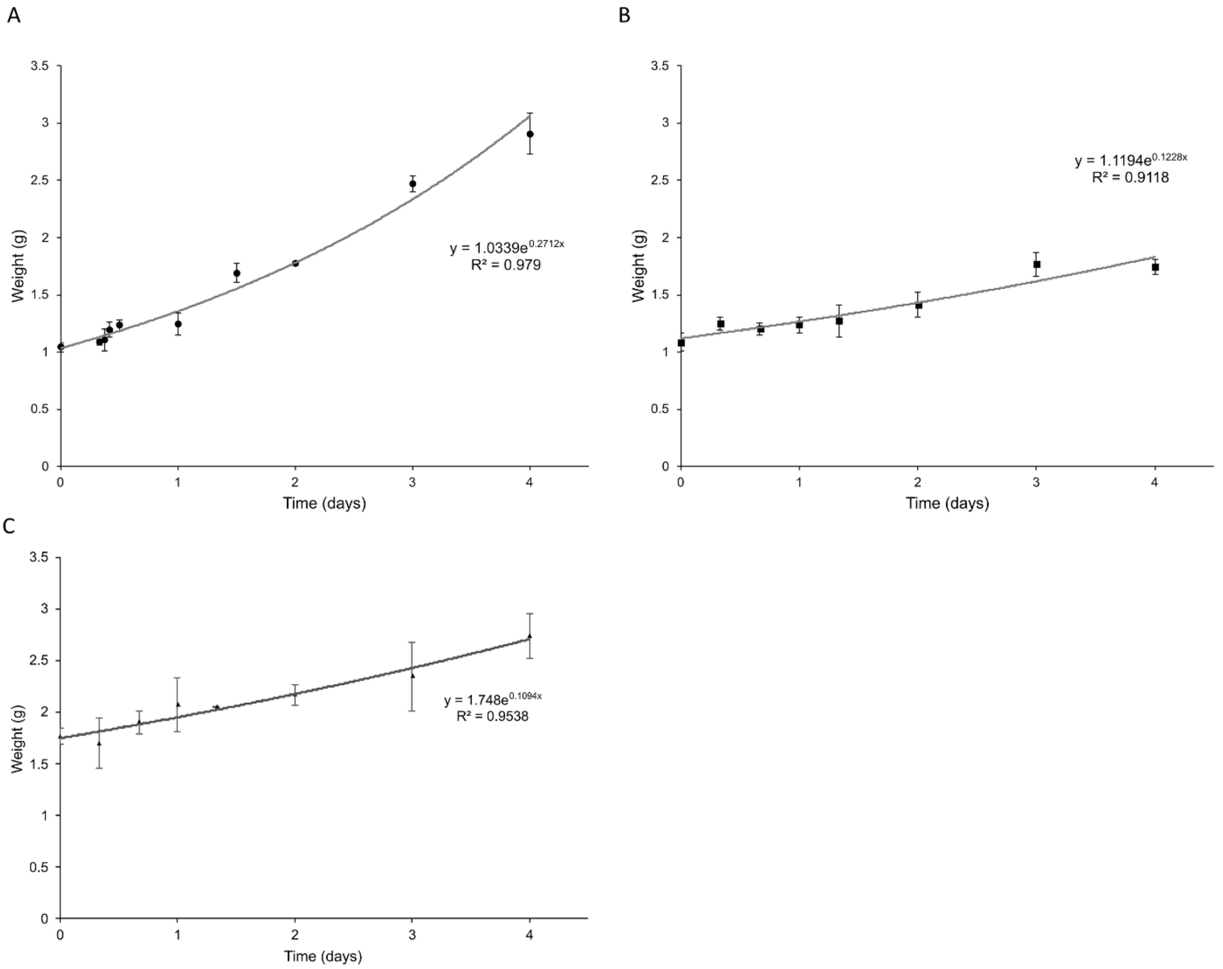

Supplemental figure 1: Seedling growth in culture. A. Col-0 optimal growth conditions (homeostasis). B. Col-0 flg22 replete medium (1 µM in medium). C. myc234 flg22 replete medium.

Supplemental figure 2

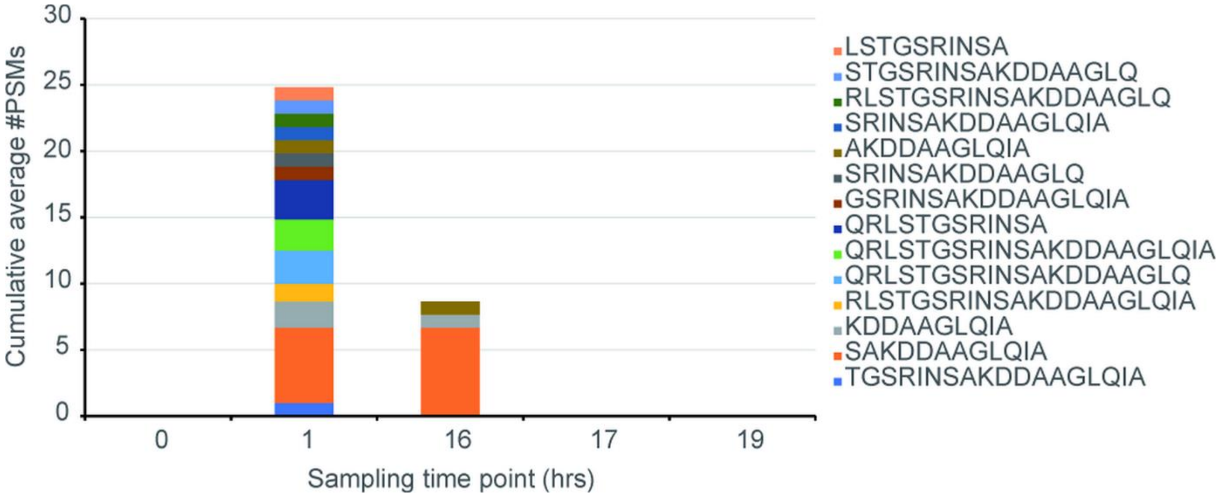

Supplemental figure 2: Flg22 full length and degradation products quantified at the 1, 16, 17 and 19 hrs sampling points (17 and 19 hrs represent 1 and 3 hrs post switch to flg22 free medium). Mean number of peptide spectral matches (#PSMs) (number of MS2 spectra annotated with a particular sequence) n=3 was used as PQI.

Supplemental figure 3

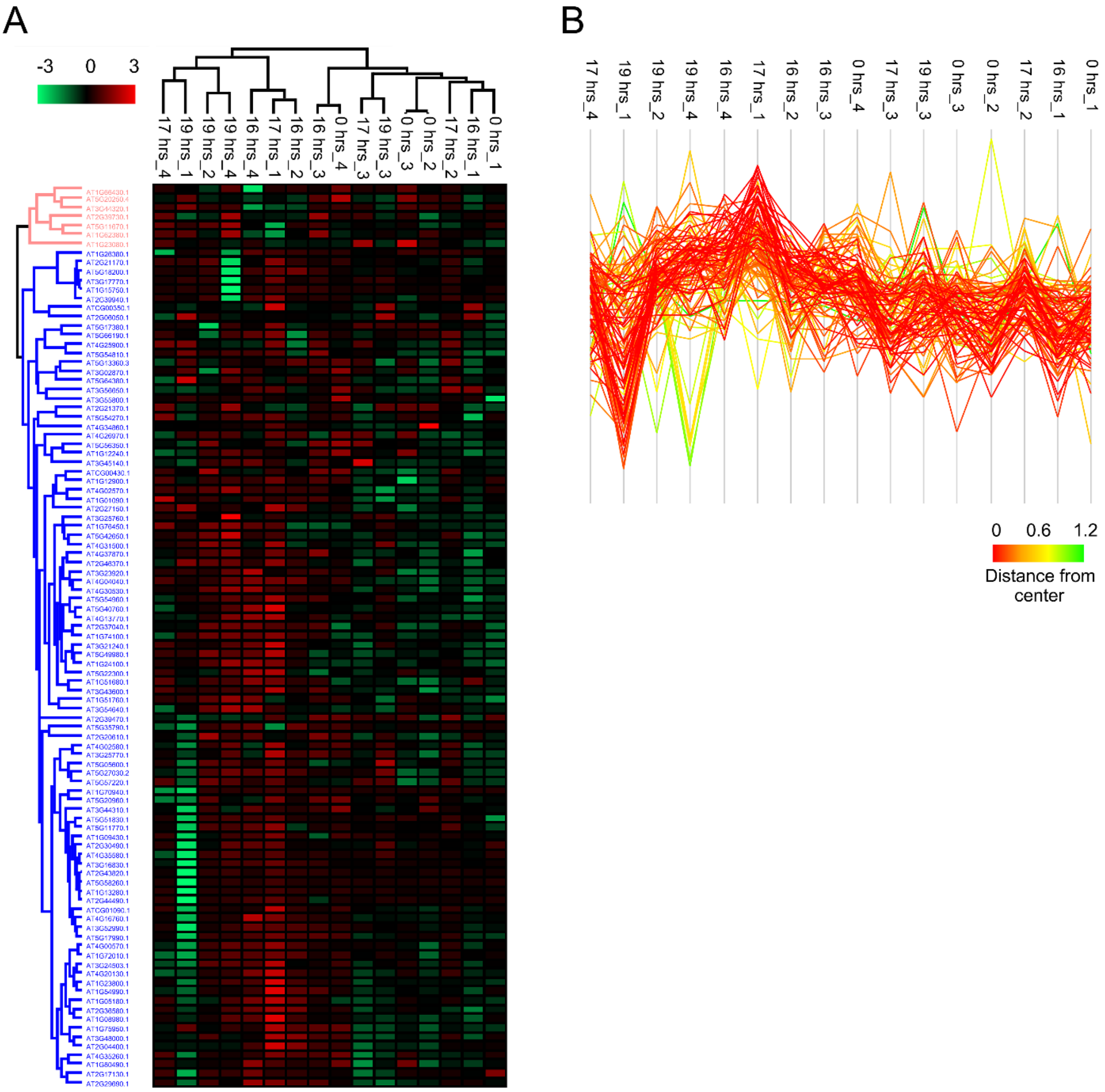

Supplemental figure 3: PRM based quantification of target proteins at 0, 16, 17 and 19 hrs sampling timepoints however without addition of flg22 to the medium at 0 hrs. Plants were transferred to new medium at the 16 hrs time point. A. Hierarchical cluster analysis of z-score transformed PQI values. B. Profile of larger blue cluster in A (n=92).
